# Administration of Once-daily Canagliflozin to a Non-diabetic Patient in Addition to Standard Aerobic Exercise: A Case Report

**DOI:** 10.7759/cureus.4352

**Published:** 2019-04-01

**Authors:** Sayak Roy

**Affiliations:** 1 Internal Medicine, Calcutta Medical Research Institute Hospital, Kolkata, IND

**Keywords:** canagliflozin, sglt2i

## Abstract

There is no Indian data at present on sodium-glucose cotransporter 2 (SGLT2) inhibitors' role on glycated haemoglobin A1c (HbA1c), weight, and blood pressure in non-diabetic individuals. This novel mechanism of action could assure us of sustained non-glycaemic benefits along with information on the negligible risk of hypoglycaemia. The aim was to observe the changes of various parameters using canagliflozin (300 mg) in a non-diabetic person suffering from hypertension and dyslipidaemia (on treatment for two years). Canagliflozin (300 mg) once-daily was administered for 13 weeks with a continuous glucose monitoring system (CGMS) installed to assess glycaemic changes and tests done at baseline: four, eight, and 13 weeks. A dyslipidaemic and hypertensive with a family history of type 2 diabetes (T2D) (mother) and hypertension (father), the patient was currently using antihypertensive and statin therapy for two years. Over a period of 13 weeks, there was a reduction in weight by 3.2 kg; body mass index (BMI) by 1 Kg/m^2^, visceral fat by 1.5%, waist circumference by 5 cm, uric acid level by 63.01%, and increase in bone mineral density (BMD) (as opposed to decrease seen with SGLT2Is in other studies). There was no episode of hypoglycaemia. Our study has given rise to certain critical issues regarding the early use of canagliflozin (although on an off-label basis) in patients who are at high risk of developing diabetes in the future.

## Introduction

Sodium-glucose cotransporter 2 (SGLT2) inhibitor produces transient natriuresis, diuresis, weight reduction, glycated haemoglobin A1c (HbA1c), and blood pressure in patients having type 2 diabetes (T2D) and also reduced cardiovascular mortality, the risk of hospitalization for heart failure, and the risk of renal events in patients with type 2 diabetes mellitus (T2DM) and those having established cardiovascular disease [1]. However, there is no Indian data to date and very rare data of SGLT2I in non-diabetics. The novel mechanism of action could assure of the negligible risk of hypoglycaemia with the sustained extra-glycemic benefits.

This case describes the effective treatment of weight loss, loss of visceral fat using Omron HBF-375 (Omron Inc., Tokyo, Japan) body fat analyzer and inflammatory markers using canagliflozin (300 mg) in a non-diabetic patient suffering from hypertension and dyspilidemia (on treatment for two years).

## Case presentation

Daily 300 mg of canagliflozin was taken by the patient from May 2018 to August 2018 over a period of 13 weeks to assess the changes with this therapy on a battery of tests as mentioned in Table 1.

**Table 1:** Description of parameters analysed during the study Hba1c:glycated haemoglobin A1c; HDL:high density lipoprotein; LDL:low density lipoprotein; VLDL:very low density lipoprotein; Tgs:triglycerides test; Pcv:packed cell volume; EPO:erythropoietin; APO B:apolipoprotein B; HS CRP:high-sensitivity C-reactive protein; Usg:ultrasonography; BP:blood pressure; BMI:body mass index; abp:ambulatory blood pressure; SBP:systolic blood pressure; BMD:bone mineral density; Rt:right; Lt:left.

Name of parameter	Baseline results	Results after 4 weeks	Results after 8 weeks	Results after 13 weeks	Results after drug washout
Hba1c (%)	5.8			5.8	
Estimated Average Glucose (mg/dl)	120			120	
Creatinine (mg/dl)	0.9			1.0	0.8
Uric Acid (mg/dl)	7.3	4.2	5.4	2.7	
T.Cholesterol (mg/dl)	117			103	
HDL (mg/dl)	31			32	
LDL (mg/dl)	52			53	
VLDL (mg/dl)	34			18	
Tgs (mg/dl)	271			167	
Hemoglobin (gm%)	13.5	13.9	14.3	14.0	13.4
Pcv (%)	42.9	43.1	44.2	42.7	41.3
EPO (miu/ml)	14.7	15	14.4	13.9	14.0
APO B (mg/dl)	68	64	60	65	
HS CRP (mg/L)	0.8			0.67	
Renal doppler resistive index (ri)					
Right renal artery hilum	0.64	0.62	0.72	0.69	
Right intra-renal artery	0.6	0.68	0.75	0.70	
Leftt renal artery hilum	0.7	0.69	0.73	0.69	
Left intra-renal artery	0.68	0.7	0.76	0.66	
Shear wave elastography of liver (kpa)					
Average	5.6			4.1	
Usg upper abdomen	Mild fatty liver			Mild fatty liver	
BP (mm Hg)	110/82			108/82	
WEIGHT (kg)	87.6	86.2	84.6	84.4	
BMI (kg/m^2^)	26.3	25.7	25.3	25.2	
Total body fat %	25.8	23.4	25.3	22.8	
Subcutaneous (sc) fat whole body %	18.1	16.5	17.6	16.1	
Sc fat trunk %	16.4	14.9	15.8	14.5	
Sc fat arm %	24.6	21.9	24.4	21.4	
Sc fat legs %	24.6	21.8	24.4	21.2	
Visceral fat %	11	10	9.5	9.5	
Sketetal muscle (sm) whole/body %	32.1	33.3	32.3	33.5	
Sm trunk %	24.5	26.1	24.7	26.4	
Sm arms %	36.5	37.5	36.9	37.7	
Sm legs %	49.4	50.6	49.5	50.7	
Body age	47	44	45	44	
Waist circumference (cms)	101	99.6	96.52	96	
Central abp					
Central SBP	105		102	102	
Pulse wave velocity	5.3		5.3	5.3	
Pulse pressure	35		37	34	
BMD gm/cm^2^					
Rt femur neck	1.089			1.096	
Lt femur neck	1.117			1.152	
Ls spine	1.444			1.485	
T-score					
Rt femur neck	0.1			0.2	
Lt femur neck	0.4			0.6	
Ls spine	1.9			2.2	

Baseline drugs used

Being a hypertensive and dyslipidemic and having a family history of T2D (mother) and hypertension (father), the patient was currently using prazosin XL (extended release) 5 mg, telmisartan 80 mg, rosuvastatin 10 mg, nevibolol 5 mg (all taken once a day) regularly for the last two years.

Drug administered 

Medtronic continuous glucose monitoring system (CGMS) (Medtronic Minimed, Northridge, CA, USA) machine was installed on 18th May and the first dose of canagliflozin 300 mg was taken at 12 pm on 20th May, 2018.

Results

**Figure 1:**
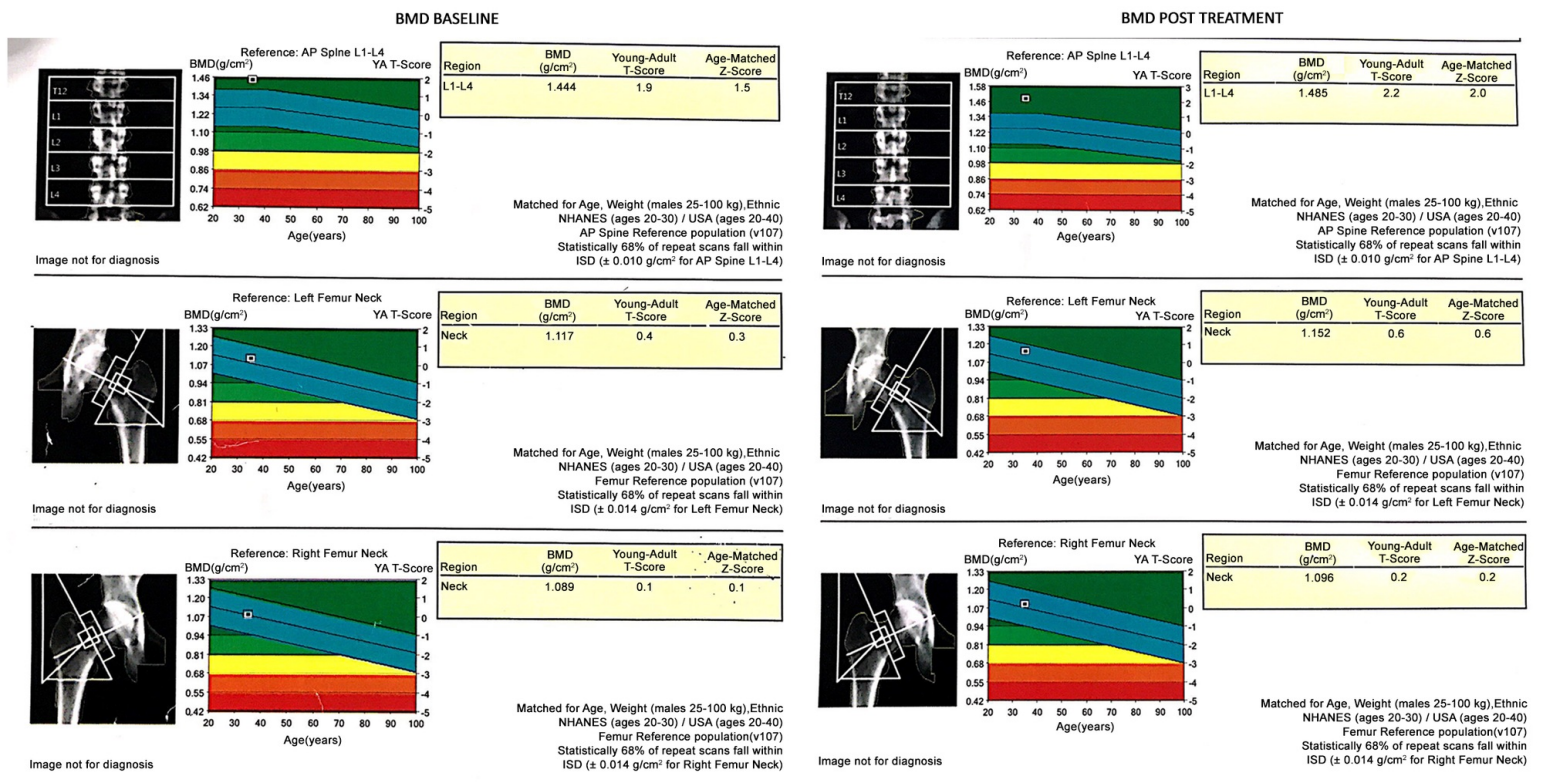
Bone mineral density (BMD) at baseline and on 13th week

**Figure 2:**
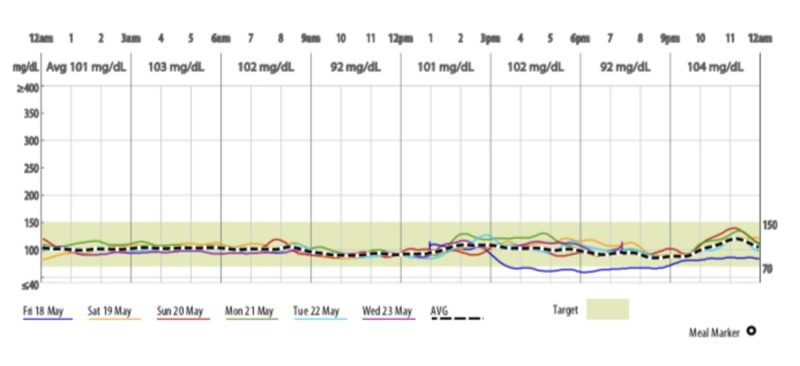
Continuous glucose monitoring system data assessed over 13 weeks

Over a period of 13 weeks, there was a reduction in weight by 3.2 kg, body mass index (BMI) by 1 Kg/m2, visceral fat by 1.5%, waist circumference by 5 cm, uric acid level by 63.01%, and mild increase in bone mineral density (BMD) as seen in Figure 1 (as opposed to decrease seen with SGLT2Is in other studies). There was no hypoglycaemia (Figure 2).

**Figure 3:**
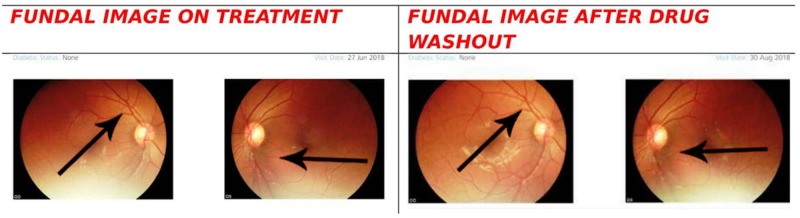
Fundal scan after one month of therapy and 10 days after drug wash out period

There was also a small drop in erythropoietin level at the end of the study (as opposed to a rise seen with SGLT2Is on T2DM in other small studies) [2]. There was no change in the fundal scan as measured by the Carl Zeiss (Visuscout 10, Zeiss, Jena, Germany) machine (Figure 3). Sleeping time (7 hours) ABPM showed a decreased trend during the drug intake period when compared with ABPM reading after 10 days of drug washout period (Table 2).

**Table 2:** Changes in sleeping (seven hours) ambulatory blood pressure monitoring (ABPM) Avg: average; SBP: systolic blood pressure; DBP: diastolic blood pressure.

Measurement done	AVG SBP	AVG DBP	AVG pulse rate	AVG mean arterial pressure	AVG pulse pressure
On treatment	101.6	60.3	71	69.8	41.33
After drug washout	103.7	74.0	62.44	76.33	36.88

## Discussion

There was also a small drop in erythropoietin level at the end of the study (as opposed to a rise seen with SGLT2Is on T2DM in other small studies) [2]. There was no change in the fundal scan as measured by Carl Zeiss - Visuscout 100 machine (Figure 3). Sleeping time (seven hours) ABPM showed a decreased trend during the drug intake period when compared with ABPM reading after 10 days of drug washout period (Table 2).

SGLT2Is have shown multiple modes of action to bring out the cardiovascular and renal benefits without causing dyselectrolytemia [3] or any serious adverse events in most of the cases. They have also shown to reduce visceral fat and increase skeletal muscle trunk [4]. Canagliflozin has provided clinically significant body-weight reductions as well as reductions in HbA1c and SBP [5] in diabetics but data in non-diabetics is insufficient. Our study showed a reduction in weight, BMI and waist circumference over the 13 weeks period.

Taking into consideration the genotypical predisposition of Asians to high cardiovascular disease (CVD) burden, the World Health Organization (WHO) expert panel has set 22.9 kg/m2 as the upper cut-off value of BMI for Asians [6].

Fracture risk in CANVAS trial (Trial number NCT01032629) was increased in patients having:

· Increased age with a previous history or having a risk of CVD or

· Having lower estimated glomerular filtration rate at baseline or

· Having more baseline diuretic use

Fractures occurred more at non-vertebral sites (at upper extremities) [7]. However, there was no increase in fracture risk in our study (instead there was a small rise in BMD), though the duration of exposure could be considered less.

The reduction of uric acid reduction by SGLT2Is has been attributed to the increased glucose delivery to distal tubules where GLUT9 absorbs that in exchange of uric acid. This action seems to persist in non-diabetics as well [7].

Increased Hematocrit level in EMPAREG outcome (Trial number NCT01131676) has been attributed to be a major cardiovascular benefit contributing factor [8]. Recently Sano et al. have proposed that the increase in erythropoietin (EPO) levels with SGLT2Is could be due to the recruitment of neural-crest-derived fibroblasts producing EPO which stops getting converted to myofibroblasts due to the reduction in proximal tubular oxygen consumption [9]. Another proposed mechanism is hypoxia at the level of corticomedullary junction due to afferent arteriolar constriction by SGLT2Is. In this case, we saw a declining trend of EPO with time which reverted back (incompletely) on stoppage of the drug. This is a new finding as SGLT2I by virtue of their afferent arteriolar vasoconstriction should actually increase in the initial stage followed by maintenance of the previous level of EPO but we found a decreasing trend, the reason of which needs to be further clarified in large scale trials.

## Conclusions

This experimental case has given rise to certain critical issues regarding the early use of canagliflozin and SGLT2Is (although on an off-label basis) in patients who are having a high risk of developing diabetes in the future. A personalized approach in specific populations is the need of the hour to control the diabetes epidemic. The persistence of these extra-glycemic benefits in non-diabetics might open up a new horizon for research with these molecules in patients of polycystic ovarian disease, nonalcoholic fatty liver disease/nonalcoholic steatohepatitis (NAFLD/NASH) where weight loss is the core-stone of therapy.
